# Depth‐Dependent Post‐Treatment for Reducing Voltage Loss in Printable Mesoscopic Perovskite Solar Cells

**DOI:** 10.1002/advs.202206331

**Published:** 2023-01-22

**Authors:** Xufeng Xiao, Wenhao Zhang, Jiale Liu, Jiankang Du, Cheng Qiu, Ranjun Meng, Anyi Mei, Hongwei Han, Yue Hu

**Affiliations:** ^1^ Wuhan National Laboratory for Optoelectronics Huazhong University of Science and Technology Wuhan 430074 China

**Keywords:** hole‐conductor‐free, mesoscopic, perovskite solar cells, printable, voltage loss

## Abstract

The printable mesoscopic perovskite solar cells consisting of a double layer of metal oxides covered by a porous carbon film have attracted attention due to their industrialization advantages. However, the tens‐of‐micrometer thickness of the triple scaffold leads to a challenge for perovskite to crystallize and for the charge carriers to separate and travel to the electrode, which limits the open circuit voltage (*V*_OC_) of such devices. In this work, a depth‐dependent post‐treatment strategy is demonstrated to synergistically passivate defects and tune interfacial energy band alignment. Two thiophene derivatives, namely 3‐chlorothiophene (3‐CT) and 3‐thiophene ethylenediamine (3‐TEA), are selected for the post‐treatment. Energy‐dispersive X‐ray spectroscopy proves that 3‐CT is uniformly distributed throughout the triple scaffold and effectively passivates the defects of the bulky perovskite, while 3‐TEA reacts rapidly with the loose perovskite in the carbon layer to form 2D perovskite, forming a type II energy band alignment at the perovskite/carbon interface. As a result, the defect‐assisted recombination is suppressed and the interfacial energy band is regulated, increasing the *V*_OC_ to 1012 mV. The PCE of the devices is enhanced from 16.26% to 18.49%. This depth‐dependent post‐treatment strategy takes advantage of the unique structure and provides a new insight for reducing the voltage loss.

## Graphical abstract

Two thiophene derivatives, namely 3‐cthlorothiophene and 3‐thiophene ethylenediamine, are selected for the post‐treating the printable mesoscopic solar cells. The different properties of the thiophene derivatives allow them to stay at different depths of the device and play different roles. Ultimately, the defect‐assisted recombination is suppressed and the interfacial energy band is regulated. The resulting devices exhibit improved performance.

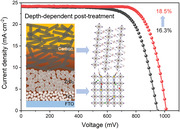

## 1 Introduction

The power conversion efficiency (PCE) of perovskite solar cells (PSCs) soared to 25.7% in just a few years,^[^ ^1^ ^]^ and the key to skyrocketing PCE is the excellent optoelectronic properties of the organic–inorganic lead halide perovskite materials.^[^ ^2^, ^3^ ^]^ The perovskite films can be fabricated by simple solution processes using earth‐abundant materials, showing potential for low‐cost photovoltaic applications.^[^ ^4^ ^]^ Among various cell structures, the printable mesoscopic PSCs are fabricated by depositing perovskite in screen‐printed mesoporous titanium dioxide (m‐TiO_2_)/mesoporous zirconium dioxide (m‐ZrO_2_)/porous carbon scaffolds,^[^ ^5^ ^]^ showing unique advantages in terms of cost, stability and large‐scale industrial fabrication.^[^ ^6^, ^7^ ^]^

Nevertheless, PCE of the printable mesoscopic PSCs is still lower than that of traditional structure devices. The main methods of improving the performance of mesoscopic PSCs include tuning the perovskite crystallization, adjusting the composition of perovskite,^[^ ^8^, ^9^, ^10^, ^11^, ^12^ ^]^ modifying the interfaces and developing the charge selective materials and electrode materials.^[^ ^13^, ^14^, ^15^, ^16^, ^17^ ^]^ Through these efforts, the highest certified PCE for the printable mesoscopic PSCs has now reached 17.7%^[^ ^12^ ^]^ and the highest reported PCE is 18.8%.^[^ ^10^ ^]^ At present, the crystallization process of perovskite in mesoscopic scaffolds is controllable, and the morphology and crystallinity have been significantly improved.^[^ ^8^, ^9^, ^10^ ^]^ This inspires us to focus more on the property tuning of perovskite itself. The voltage loss of printable mesoscopic PSCs is more severe than the traditional structure PSCs, which can be overcome by reducing the defect density and non‐radiative recombination losses.^[^ ^18^, ^19^ ^]^ Some defects with deep energy levels such as uncoordinated Pb^2+^, Pb–I anti‐substitution, and Pb clusters are easily generated at the grain boundaries and interfaces of perovskite, leading to defect‐assisted non‐radiative recombination.^[^ ^20^, ^21^ ^]^ The small size perovskite grains and many grain boundaries in the printable mesoscopic structure may lead to the generation of more defects with deep energy levels. In addition, the interface recombination caused by mismatched energy level alignment, interfacial defect, and carrier back transfer can also severely impair the *V*_OC_ of the printable mesoscopic devices.^[^ ^22^ ^]^

The unclear interface between the perovskite and the functional layers in the mesoscopic structure leads to many strategies of defects passivation and interface treatment unsuitable for the printable mesoscopic PSCs. Moreover, the multiple strategies used in succession to passivate defects or tune interface can easily lead to process incompatibility and impair device photovoltaic performance. Therefore, a strategy that can simultaneously passivate defects and tune the perovskite/carbon electrode interface is of great interest. The perovskite in m‐TiO_2_/m‐ZrO_2_ and porous carbon electrodes are significantly different due to the difference in the pore sizes and hydrophilicity of the scaffold materials.^[^ ^15^, ^17^ ^]^ In m‐TiO_2_/m‐ZrO_2_, the perovskite grains are tightly filled in the mesoscopic scaffolds without pinholes. In porous carbon electrodes, the perovskite is neither tightly bonded with the carbon black particles nor graphite flakes, and the morphology of perovskite is loose and porous. Through rational selection of functional additives, we are able to achieve different perovskite properties in different depths thanks to the various morphology in each scaffold layer.

Herein, we demonstrate a post‐treatment strategy based on thiophene derivatives, namely 3‐chlorothiophene (3‐CT) and 3‐thiophene ethylenediamine (3‐TEA) to passivate the perovskite defects in m‐TiO_2_/m‐ZrO_2_ and in the meantime to tune perovskite/carbon interface. We found the smaller size 3‐CT distributed throughout the triple scaffold while the larger size 3‐TEA reacts rapidly with the loose perovskite to form 2D perovskite in the carbon layer. As a result, the 3‐CT/3‐TEA co‐treatment strategy effectively reduces the defect‐assisted recombination, regulates the interfacial energy levels, prevents the carrier back transport, and reduces the interfacial recombination. This increases the *V*_OC_ of the device from 950 to 1012 mV and PCE from 16.26% to 18.49%.

## 2 Results and Discussion

**Figure 1:**
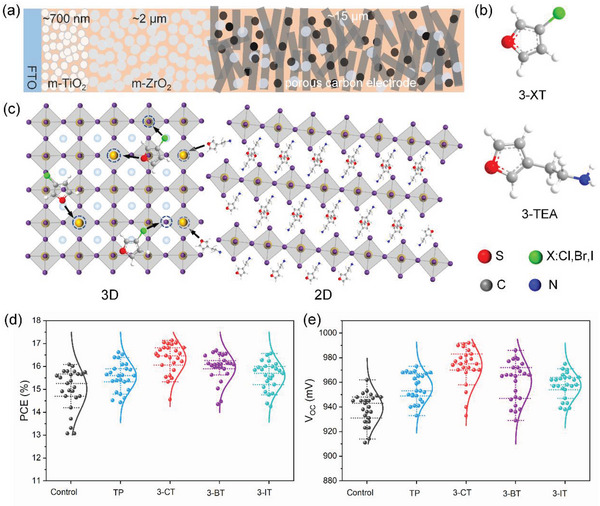
a) Schematic diagram of the printable mesoscopic PSCs structure. b) Molecular structures of 3‐XT and 3‐TEA. c) Schematic diagram of the interaction of 3‐XT and 3‐TEA with perovskite. Box plots of d) *V*_OC_ and e) PCE of the printable mesoscopic devices treated with thiophene and its halogenated derivatives.

The schematic diagram of the device is shown in **Figure** **1**a. The thicknesses of m‐TiO_2_ and m‐ZrO_2_ are ≈700 nm and 2 µm, respectively, and the thickness of the porous carbon electrode is ≈15 µm. As shown in Figure S1 (Supporting Information), due to the different thickness, pore size and hydrophilicity of the scaffold materials, the perovskite exhibited dense and sparse porous morphologies in m‐TiO_2_/m‐ZrO_2_ and porous carbon electrode, respectively. This facilitates the selection of suitable molecules to regulate the properties of perovskite at different depths. Recently, thiophene‐based 2D perovskite exhibited better photovoltaic properties than phenylethylammonium‐based cationic 2D perovskite.^[^ ^23^, ^24^ ^]^ While forming 2D perovskite, the electron‐rich *π*‐functional group in thiophene and the stronger interaction between Pb–S can passivate the uncoordinated Pb^2+^, and the strongly polar S atom can also facilitate the charge transport.^[^ ^25^, ^26^ ^]^ Therefore, two types of thiophene molecules are applied to treat the devices, and their molecular structures are shown in Figure 1b. The halogen thiophene (3‐XT) and 3‐TEA have the potential to achieve spatially different distribution and effect due to the differences in their molecular structure. We expect that the halogen atom at the interposition of the 3‐XT will form halogen bonds with I to inhibit the formation of I defects and that S in thiophene containing lone pairs of electrons can passivate uncoordinated Pb defects in the perovskite in the whole triple scaffold. 3‐TEA with ethylamine group is expected to react with the loose and porous perovskite in carbon electrode, possibly forming wide band gap 2D perovskite and forming the type‐II energy band arrangement at the interface, inhibiting interfacial recombination. Moreover, because there is enough perovskite in the carbon electrode, most of the 3‐TEA will form 2D perovskite distributed in the carbon electrode without impairing the light absorption of the device (Figure 1c).

First, we compared the effect of different halogen‐substituted thiophenes, i.e., thiophene (TP), 3‐chlorothiophene (3‐CT), 3‐bromothiophene (3‐BT), and 3‐iodothiophene (3‐IT), in passivating perovskite defects. We treated the devices by drop coating the fabricated devices with isopropanol solutions containing different molecules and dried them naturally at room temperature. The PCE results are shown in Figure 1d,e. All four can enhance the *V*_OC_ and PCE of the devices, while the 3‐CT treatment shows the most prominent effect for *V*_OC_ and PCE enhancement. Based on the above experimental results with monosubstituted halogen‐thiophenes, 3‐CT was used as a defect passivation molecule for further experiments. In the subsequent experiments, the untreated sample and the samples treated separately with 3‐CT and 3‐TEA were named Control, 3‐CT, and 3‐TEA, respectively, and the 3‐CT and 3‐TEA synergistically treated sample was named 3‐CT/3‐TEA.

**Figure 2:**
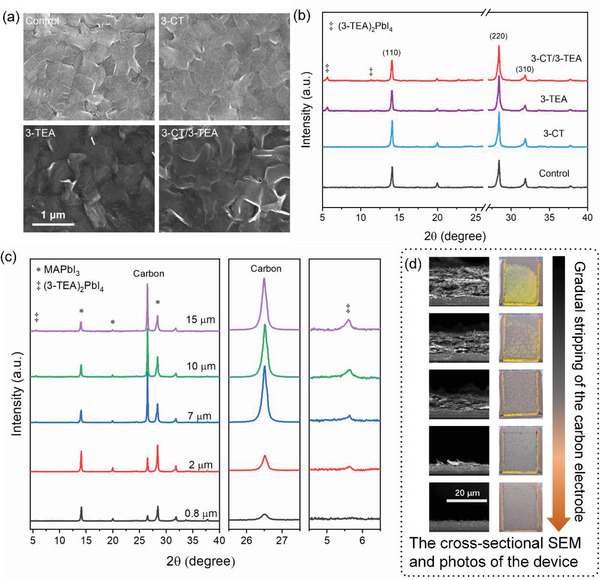
a) Surface SEM images of the different perovskite films. b) XRD patterns of the different perovskite in the devices. c) XRD patterns of 3‐TEA treated device after gradual stripping of carbon electrode. d) The cross‐sectional SEM images and photos of 3‐TEA treated device after gradual stripping of carbon electrode.

We then studied the surface and cross‐sectional morphologies of the perovskite films and devices by scanning electron microscopy (SEM). As shown in **Figure** **2**a, the surface morphologies of the 3‐CT treated perovskite film are similar to those of the control, but the boundaries between the grains of the 3‐TEA and 3‐CT/3‐TEA treated perovskite films became blurred, and sheet‐like grains appeared, indicating that the 3‐TEA treatment may lead to the formation of 2D perovskite on the surface of the films. The morphologies of the 3‐CT and 3‐TEA treated devices are shown in Figure S2 (Supporting Information). The surface and cross‐sectional morphologies of the 3‐CT treated devices are not significantly different from those of the control. Notably, the surface of the device turns yellow after 3‐TEA treatment. The surface and cross‐sectional SEM images illustrate that the yellow substance exhibits a sheet‐like structure and is distributed on the surface of the carbon electrode.

The crystal structure of perovskite in the devices after the treatment was investigated by X‐ray diffraction (XRD). As shown in Figure 2b, compared with the control, 3‐CT treatment slightly increased the intensity of the characteristic peaks, indicating an improved crystallinity. This may be caused by recrystallization of perovskite and passivation of defects by the post‐treatment. The intensities of the characteristic peaks of the perovskite in the 3‐TEA and 3‐CT/3‐TEA treated devices are similar to that of the control. The XRD patterns of the devices treated with different contents of 3‐CT and 3‐TEA are shown in Figure S3 (Supporting Information). In addition, we notice diffraction peaks appearing at 2*θ* = 5.67° and 11.33° with the addition of 3‐TEA and we speculate them to be characteristic peaks of 2D perovskite formed by the reaction of 3‐TEA and MAPbI_3_. To verify this speculation, we grew a 2D perovskite single crystal with 3‐TEA as a spacer cation As shown in Figure S4 (Supporting Information), we confirmed that the obtained single crystal is Ruddlesden–Popper perovskite (3‐TEA)_2_PbI_4_.^[^ ^24^ ^]^ The (020) and (040) lattice planes of (3‐TEA)_2_PbI_4_ correspond to the characteristic peaks at 2*θ* = 5.67° and 11.34°, respectively, which are consistent with the 2D perovskite peak positions appearing in Figure 2b. This indicates that the sheet‐like structures appearing on the porous carbon electrode surface of the 3‐TEA treated device are 2D perovskite (3‐TEA)_2_PbI_4_.

The changes in surface morphology inspired us to gain further insight into the spatial distribution of 3‐CT and 3‐TEA in the treated devices, which can be determined by the distribution of S in the cross‐section of the treated devices by energy‐dispersive X‐ray spectroscopy coupled with SEM (EDX). Notably, the signal from Pb overlaps with that from S in EDX (Figure S5, Supporting Information),^[^ ^27^ ^]^ therefore, we used Sn‐based perovskite (FASnI_3_) to replace MAPbI_3_. In EDX‐SEM mapping (Figure S6, Supporting Information), the S element represents the 3‐CT or 3‐TEA molecules. The 3‐CT treated device exhibits a uniform S element distribution signal in the m‐TiO_2_/m‐ZrO_2_/porous carbon scaffolds, indicating that the 3‐CT treatment can make the 3‐CT molecule uniformly distributed in the triple‐layer scaffolds. The S signal in the 3‐TEA treated device is mainly distributed in the porous carbon layer, which indicates that 3‐TEA mainly interacts with perovskite in the porous carbon layer without entering m‐TiO_2_/m‐ZrO_2_. Since 3‐TEA can react with MAPbI_3_ to form 2D perovskite, we further investigated the distribution of 2D perovskite in the porous carbon electrode. As shown in Figure 2d, we stripped the carbon electrode layer‐by‐layer with tape and measured the corresponding carbon layer thickness by SEM. The thickness of the carbon layer was thinned from the initial 15 to 10, 7, 2, and 0.8 µm in order, which corresponded to the sample carbon layer thicknesses of the optical photographs and XRD patterns in Figure 2d,c. As the carbon layer is stripped, the 2D perovskite on the device surface gradually decreases until it disappears (Figure 2d). The intensity of the diffraction peak of carbon at 2*θ* = 26.49° also decreases gradually with the stripping of the carbon layer, while the intensity of the characteristic peak of the 2D perovskite at 2*θ* = 5.67° gradually decreases until it disappears (Figure 2c). This is strong evidence that 2D perovskite is distributed only in the porous carbon layer, agreeing with the EDX‒SEM results. This suggests that our idea of tuning different perovskite properties at different depths by selecting the appropriate functional molecules in the device is well‐realized.

**Figure 3:**
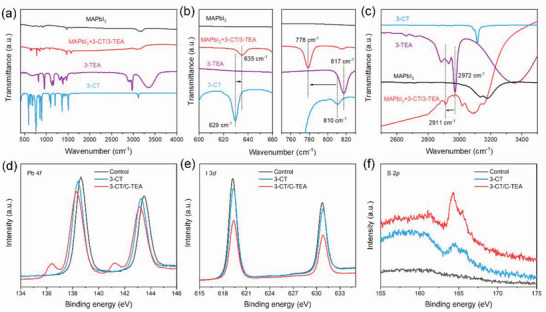
a) FT‐IR spectra of 3‐CT, 3‐TEA, MAPbI_3_ control sample, and MAPbI_3_ treated with 3‐CT/3‐TEA. Local enlarged FT‐IR spectra of b) C–Cl bond, C–S bond, and c) N–H bond. High‐resolution XPS spectra of d) Pb 4f, e) I 3*d*, and f) S 2*p*.

The interaction of the two molecules with perovskite can be investigated by Fourier transform infrared (FT‐IR) spectroscopy. The spectra of the 3‐CT/3‐TEA treated perovskite consisted of vibration peaks of MAPbI_3_, 3‐CT, and 3‐TEA, proving the effective interaction between 3‐CT, 3‐TEA, and the perovskite (**Figure** **3**a). As shown in Figure 3b, the peak at 629 cm^−1^ in the 3‐CT spectrum could be assigned to the stretching vibration of the C–Cl bond.^[^ ^28^ ^]^ In the 3‐CT/3‐TEA‐treated sample, the corresponding peak shifted to a higher wavenumber of 635 cm^−1^. This change occurred due to the formation of Cl–I halogen bonds enhanced C–Cl bond interactions. According to Mulliken's charge‐transfer model,^[^ ^29^ ^]^ the Cl–I halogen bond increases the effective charge density of C and decreases the corresponding force constant of the C–Cl bond, resulting in a redshift in the FT‐IR spectrum of the C–Cl bond. The C–S bond stretching vibrations in the thiophene ring in 3‐CT and 3‐TEA appeared at wavenumbers of 810 and 817 cm^−1^, respectively.^[^ ^30^ ^]^ In the spectra of the 3‐CT/3‐TEA‐treated perovskite film, the C–S bond peak shifted to a lower wavenumber of 778 cm^−1^. This indicates that S provides lone pair electrons and uncoordinated Pb^2+^ coordination in the perovskite. This charge transfer reduces the effective charge density of the C–S bond, resulting in a blue shift in the FT‐IR spectrum of the C–S bond. The peak of the N–H vibration in 3‐TEA appears at 2972 cm^−1^. The –NH_3_^+^ in 3‐TEA and PbI_2_ frameworks form 2D perovskite through hydrogen bonding, which leads to an increase in bond length and electron cloud density averaging of the N–H vibration, resulting in the N–H vibration shifted to lower wavenumbers of 2911 cm^−1^ (Figure 3c).^[^ ^31^ ^]^ To further investigate the passivation effect of 3‐CT and 3‐TEA on perovskite, we characterized the X‐ray photoelectron spectroscopy (XPS) of the perovskite films. Compared with the control, the Pb 4*f* peaks of the 3‐CT and 3‐CT/3‐TEA treated perovskites shifted toward lower binding energies, and the peak area also decreases regularly. This confirms that both 3‐CT and 3‐TEA can act as electron‐donating Lewis bases to passivate positively charged defects such as iodine vacancies, uncoordinated Pb^2+^, and Pb clusters by coordination. It should be noted that the 3‐CT/3‐TEA treated perovskite films show peaks at 136.5 and 141.4 eV (Figure 3d). This is usually considered as the peaks of uncoordinated Pb or PbI_2_ in perovskite being reduced to zero‐valent lead (Pb^0^) under X‐ray irradiation.^[^ ^32^ ^]^ we speculate that Pb^0^ is generated by the X‐ray reduction of the 2D perovskite on the surface of the film after 3‐TEA treatment. The Pb^0^ signals for the 3‐TEA treated perovskite and (3‐TEA)_2_PbI_4_ perovskite also prove this speculation (Figure S7, Supporting Information). The (3‐TEA)_2_PbI_4_ perovskite does not impair device stability.^[^ ^33^ ^]^ The binding energy of peaks of I 3*d* is slightly shifted to a high binding energy in the treated perovskite films compared with the control perovskite (Figure 3e). This indicates the formation of the Cl–I halogen bond and its passivation effect. The difference in the peak intensities of S 2*p* and Cl 2*p* in the treated films shows the change in the perovskite surface after treatment with 3‐CT and 3‐TEA in this way (Figure 3f; Figure S8, Supporting Information).

**Figure 4:**
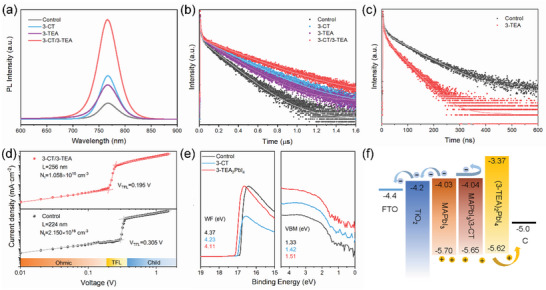
a) Steady‐state PL spectra of the perovskite films with the different molecular treatment. TRPL spectra of the perovskite films with the different molecular treatment on b) m‐ZrO_2_ and c) m‐ZrO_2_/carbon electrode. d) SCLC measurements of electron‐only devices based on the MAPbI_3_ and MAPbI_3_ treated with 3‐CT/3‐TEA. e) The UPS spectra of MAPbI_3_, MAPbI_3_ treated with 3‐CT, and (3‐TEA)_2_PbI_4_. f) The energy level diagram of the printable mesoscopic PSCs device.

Steady‐state photoluminescence (PL) was applied to further understand the positive effect of 3‐CT/3‐TEA treatment on the passivation of perovskite defects. 3‐CT, 3‐TEA, and 3‐CT/3‐TEA treated perovskite films showed significantly increased intensities compared to the control film, which indicates that 3‐CT and 3‐TEA treatments reduce the non‐radiative recombination caused by defects through passivation (**Figure** **4**a). We further confirm the above conclusion by investigating the kinetics of photogenerated carriers of perovskite by time‐resolved photoluminescence (TRPL) decay measurements. Compared with the control perovskite films (52.73 ns), the average lifetimes of the films treated 3‐CT and 3‐TEA are 90.02 and 77.84 ns, respectively, and the 3‐CT/3‐TEA‐treated perovskite film exhibits the longest carrier lifetime (136.25 ns). The difference in carrier lifetime indicates that both 3‐CT and 3‐TEA can passivate defects and inhibit recombination (Figure 4b), and the passivation effect of 3‐CT/3‐TEA treatment is more significant. The detailed exponential fitting results are shown in Table S1 (Supporting Information). The space‐charge limited current (SCLC) method was applied to quantitatively evaluate the defect density of perovskite films to further confirm the passivation ability of 3‐CT/3‐TEA treatment (Equation S1, Supporting Information). As shown in Figure 4d, the defect densities in the control and 3‐CT/3‐TEA treated perovskite films are 2.150 × 10^–16^ and 1.058 × 10^–16^ cm^−3^, respectively. The 3‐CT and 3‐TEA treated perovskite films exhibited defect densities of 1.440 × 10^–16^ and 1.954 × 10^–16^ cm^−3^, respectively (Figure S9, Supporting Information). This can be explained by the different distributions of these thiophene additives. The 3‐CT/3‐TEA treated devices exhibit the lowest defect density and the best passivation due to the different distribution and passivation pathways of 3‐CT and 3‐TEA.

The in situ generation of a wide band gap 2D perovskite at the contact interface between the perovskite and carbon electrode can effectively block the diffusion of electrons to the carbon electrode and inhibit interfacial recombination. The absorbance of the 3‐CT treated perovskite film increased slightly due to the increase in crystallinity, and the band gap did not change (Figure S10, Supporting Information). The 3‐CT/3‐TEA treated perovskite film exhibits a slightly reduced absorbance compared to the control and some absorption cutoff edges located before 700 nm, indicating that the 2D perovskite generated on the surface affects the light absorption of the perovskite films. We further analyzed the *n*‐value of the 2D perovskite formed on the surface of the film (Figure S11, Supporting Information). UV‒vis spectra of treated devices without C electrode demonstrated that the 2D perovskite formed by the 3‐CT/3‐TEA treatment does not impair the light absorption of the devices (Figure S12, Supporting Information). The band gaps of the perovskite films and 2D perovskite are obtained from Tauc plots (Figure S13, Supporting Information) The work function and the valence band maximum (VBM) were characterized by ultraviolet photoelectron spectroscopy (UPS) (Figure 4e). The energy level diagram of the printable mesoscopic PSCs is shown in Figure 4f. The pristine perovskite MAPbI_3_ and 2D perovskite (3‐TEA)_2_PbI_4_ form type‐II energy band arrangement, which can effectively block the electron back transport to the carbon electrode and inhibit interfacial recombination. In addition, we also evaluated the effect of type II band alignment on carrier extraction at the perovskite/carbon electrode interface by TRPL (Figure 4c). The detailed fitting parameters are shown in Table S2 (Supporting Information). The average lifetimes of the control and 3‐TEA treated sample are 26.14 and 12.48 ns, respectively. The reduced carrier lifetime can be attributed to the improved extraction of holes by the carbon electrode due to the type II energy band alignment.

**Figure 5:**
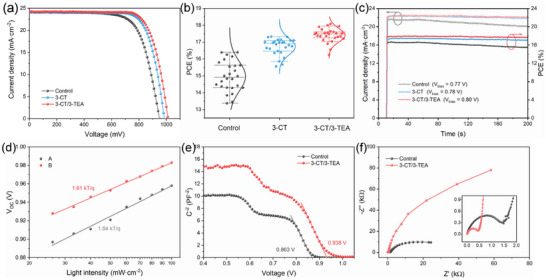
a) The *J–V* curves of the champion devices. b) Box plot of different devices PCE distribution and the statistical data were collected from 25 devices each. c) Stabilized PCEs of the devices measured at a bias of *V*_max_ under 100 mW cm^−2^ AM 1.5 G irradiation. d) The *V*_OC_ response of devices with varied light intensities. e) Mott−Schottky plots of devices. f) Nyquist plots of the devices without external bias under dark condition.

To investigate the effect of 3‐CT/3‐TEA treatment on the photovoltaic performance of PSCs, we prepared PSCs based on printable mesoscopic structures. The *J–V* curves of the devices are shown in **Figure** **5**a. Compared to the control devices with 950 mV of *V*_OC_, the devices with 3‐CT and 3‐CT/3‐TEA treatment obtained 984 and 1012 mV of *V*_OC_, respectively, and the PCE of the corresponding champion devices increased from 16.26% of control to 17.76% and 18.49%, respectively. The improvement in cell performance is mainly due to post‐treatment for passivation of defects and tuning of interfacial properties in mesoscopic cells. The statistics of the device performance parameters are shown in Figure 5b and Figure S14 (Supporting Information). The *J–V* curve of the 3‐TEA post‐treated device is shown in Figure S15 (Supporting Information). We also explored the effect of different concentrations of 3‐CT and 3‐TEA treatments on the device performance. The *V*_OC_ of the device increased with increasing 3‐CT concentration, while the *J*_SC_ of the device did not change much (Figure S16, Supporting Information). The devices already treated with optimized 3‐CT were subjected to 3‐TEA treatment to achieve synergy between 3‐CT and 3‐TEA. The *V*_OC_ of the 3‐CT/3‐TEA‐treated devices was further enhanced to 1020 mV. However, the higher concentration of 3‐TEA treatment may impair the absorption of perovskite, leading to a decrease in the *J*_SC_ of the devices (Figure S17, Supporting Information). The stabilized power output (SPO) was determined by measuring the current at a fixed maximum power point (MPP) voltage over 200 s. The SPO of the 3‐CT and 3‐CT/3‐TEA treated devices reached 18% and 17.3%, respectively, and showed better stability than the control (Figure 5c). The incident photon‐to‐current efficiency (IPCE) spectra of different devices are shown in Figure S18 (Supporting Information). The 3‐CT and 3‐CT/3‐TEA treated devices exhibit integrated currents of 22.5 and 22.3 mA cm^−2^, respectively, which are similar to that of the control. The integrated current from the IPCE is slightly lower than the photocurrent obtained from the *J–V* curves, which is a common phenomenon in printable mesoscopic solar cells. Our previous study suggested that this is related to the light soaking effect.^[^ ^12^, ^34^ ^]^

To further investigate the carrier behavior in the device, we measured the light‐dependent *V*_OC_ (Figure 5d). The defect‐assisted non‐radiative complex can be analyzed by the ideal factor (Equation S2, Supporting Information).^[^ ^35^ ^]^ The ideal factors of control device and 3‐CT/3‐TEA treated device are 1.84 and 1.61, respectively, which indicates that the co‐treatment strategy can effectively reduce the non‐radiative recombination losses in the devices. The linear variation of photocurrent with light intensity indicates an unblocked carrier transport in the device. We further measured the C–V data of the device and analyzed the carrier transport with the Mott−Schottky equation (Equation S3, Supporting Information).^[^ ^36^ ^]^ The built‐in potentials of the control and 3‐CT/3‐TEA treated devices fitted by this equation are 0.863 and 0.938 V, respectively (Figure 5e). The elevated built‐in potential facilitates the separation, transport, and extraction of carriers. the built‐in potential of the 3‐CT treated device is 0.910 V, which is slightly lower than that of the 3‐CT/3‐TEA treated device (Figure S19, Supporting Information). This indicates that 3‐CT/3‐TEA can facilitate the separation and extraction of charges in the devices more effectively. The Nyquist plots of the devices are shown in Figure 5f and Figure S20 (Supporting Information). Compared with the control, the 3‐CT/3‐TEA treated devices exhibit smaller charge transfer resistance, indicating the improved charge transfer. In addition, owing to the passivation of defects and modulation of the interface by 3‐CT/3‐TEA treatment, the non‐radiative recombination is effectively suppressed and the recombination resistance of the 3‐CT/3‐TEA treated devices is significantly increased.

To investigate the applicability of the post‐treatment strategy, we fabricated printable mesoscopic PSCs using Cs_0.05_MA_0.15_FA_0.8_PbI_3_ as the absorbing layer. As shown in Figure S21 (Supporting Information), the 3‐CT post‐treatment effectively increases the *V*_OC_ of the devices. However, the *J*_SC_ and FF of the devices are reduced after 3‐TEA treatment. This may be caused by the reaction between 3‐TEA and FA‐based perovskite resulting in the generation of *δ*‐phase perovskite, which impairs the light absorption of the devices. We suggest replacing 3‐TEA with ammonium molecules such as phenylethylammonium and pentafluorophenylethylammonium that do not destroy FA‐based perovskite for post‐treatment to achieve the goal of modulating interfacial properties.^[^ ^17^ ^]^

To investigate the effect of post‐treatment on the stability of perovskite, we measured the XRD patterns of the devices to characterize the crystal changes of perovskite at 85 °C in N_2_ atmosphere (Figure S22, Supporting Information). After 230 h of storage, the perovskites of the 3‐CT, 3‐TEA and 3‐CT/3‐TEA treated devices exhibited higher peak intensity of MAPbI_3_ (110) crystal facet and lower peak intensity of PbI_2_, indicating that the post‐treatment does not impair the stability of the perovskites. We checked the hysteresis of the devices. Neither the control nor the 3‐CT/3‐TEA treated devices showed serious hysteresis, which indicates that the post‐treatment does not exacerbate ion migration. (Figure S23, Supporting Information). We also evaluated the long‐term storage stability of the devices. The unencapsulated devices were stored in air at room temperature and 50 ± 20%RH humidity. As shown in Figure S24 (Supporting Information), due to effective defect passivation, the 3‐CT/3‐TEA treated devices maintained 94% of the peak PCE, after over 2800 h of storage.

## 3 Conclusion

In summary, based on the molecule design and the morphology difference of perovskite in the triple‐layer scaffold, we developed a strategy to reduce the non‐radiative recombination of the printable mesoscopic device. 3‐CT can penetrate the perovskite film and passivate the defects in the triple‐layer scaffold. 3‐TEA can react rapidly with the loose porous MAPbI_3_ perovskite in carbon electrode to form 2D perovskite, which can effectively regulate the perovskite/carbon electrode interface and form type‐II energy band arrangement with MAPbI_3_ to block the electron back transport. The 3‐CT/3‐TEA treated devices exhibit reduced defect density, improved charge transport, and minimized non‐radiative complexation. As a result, the *V*_OC_ of the device is raised to 1012 mV and the PCE reaches 18.49%. Our work provides a new perspective for reducing the non‐radiative recombination of the printable mesoscopic devices, which is beneficial for further improving the device photovoltaic performance.

## Conflict of Interest

The authors declare no conflict of interest.

## Supporting information

Supporting Information

Supporting Information

## Data Availability

The data that support the findings of this study are available in the supplementary material of this article.
