# Supplementary material for: Depth‐Dependent Post‐Treatment for Reducing Voltage Loss in Printable Mesoscopic Perovskite Solar Cells
Source: Adv Sci (Weinh). 2023 Jan 22;10(9):2206331. doi: 10.1002/advs.202206331 (PMC10037989; doi:10.1002/advs.202206331)
Supplement: Supplementary file 1 — Supporting Information [file ADVS-10-2206331-s001.pdf]

ADVANCED SCIENCE
Open Access

Supporting Information

for *Adv. Sci.*, DOI 10.1002/advs.202206331

Depth-Dependent Post-Treatment for Reducing Voltage Loss in Printable Mesoscopic Perovskite Solar Cells

*Xufeng Xiao, Wenhao Zhang, Jiale Liu, Jiankang Du, Cheng Qiu, Ranjun Meng, Anyi Mei, Hongwei Han and Yue Hu**

Depth-dependent post-treatment for reducing voltage loss in printable mesoscopic perovskite solar cells

Xufeng Xiao[a], Wenhao Zhang[a], Jiale Liu[a], Jiankang Du[a], Cheng Qiu[a], Ranjun Meng[a], Anyi Mei[a], Yue Hu[a,*], and Hongwei Han[a,*].

[a]Wuhan National Laboratory for Optoelectronics, Huazhong University of Science and Technology, Wuhan 430074, China.

Experimental Section

Material: Lead (II) iodide (PbI_2) was purchased from TCI. Methylammonium iodide (MAI) and methylamine hydrochloride (MACl) were purchased from MaterWin New Materials. 3-chlorothiophene (3-CT) and 3-iodothiophene (3-IT) were purchased from Aladdin. Thiophene (TP), 3-bromothiophene (3-BT), 2-(Thiophen-3-yl) ethanamine hydrochloride (3-TEACl), Hydroiodic acid (HI, 55.0-58.0 wt% in H_2O), Hypophoaphoeous acid (H_3PO_2, 50 wt% in H_2O) were purchased from Macklin. N,N-dimethylformamide (DMF), dimethyl sulfoxide (DMSO), isopropanol (IPA), Lead oxide (PbO), titanium diisopropoxide bis(acetylacetonate) (75 wt% in IPA), and terpineol were purchased from Sigma-Aldrich. Titanium dioxide paste (TiO_2, NR30) was purchased from GreatCell Solar. All the materials were used as received without further purification.

Perovskite precursor solution preparation: 0.2766g PbI_2, 0.0954g MAI, and 0.0081g MACl were added sequentially to the mixed solvent DMF/DMSO (400 μL/100 μL), and then stirred at 60 °C for 12 hour.

Growth of $(3\text{-TEA})_2PbI_4$ single crystal: 0.4464g PbO was added to a mixed solution of 6mL HI and 0.5mL H_3PO_2, heated and stirred at 60 °C until complete dissolution, and then 0.32734g 3-TEACl was added the clarified solution, heated and stirred continuously at 100 °C for 6h and then cooled naturally to room temperature, and the crystallized orange crystals are filtered and dried to obtain $(3\text{-TEA})_2PbI_4$ single crystals.

Device fabrication: The FTO glass was first laser etched to get the electrode pattern, then cleaned with detergent, deionized water and ethanol ultrasonically in turn. The dense layer was deposited on the FTO by spray pyrolysis of titanium diisopropoxide bis(acetylacetonate) at 450 °C. The m-TiO_2 was fabricated by screen-printing using diluted titanium dioxide paste (1:5 in terpineol) onto the dense layer and sintered at 500°C in air for 40 min. The m-ZrO_2 and porous carbon electrode were sequentially fabricated by screen-printing onto m-TiO_2 and sintered at 400 °C for 40 min. After the triple-layer scaffold cooling to room temperature, the precursor solution was drop-cast and penetrated in the scaffold, and then annealed at 100 °C for

30-60 min. The molar concentration of PbI_2 in the precursor solution was defined as 1. TP, 3-IT, 3-BT, 3-CT, and 3-TEACl were dissolved respectively in IPA to prepare post-treatment solutions with different percentage molar concentrations, and the fabricated devices were post-treated with these solutions. 3-CT/3-TEA treatment is to treat the device with 3-CT solution and then treat it with 3-TEACl solution immediately after the natural evaporation of IPA.

Characterization: The SEM images and EDX analysis were obtained by a NanoSEM 450 field-emission scamming electron microscope (FEI). The XRD spectra were measured with an X'pert PRO X-ray diffractometer using Cu Kα radiation under operation conditions of 40 kV and 40 mA. The UV-vis spectra were measured by a Lambda950 spectrophometer SolidSpec-3700 (PerkinElmer). The steady-state PL measurements were recorded with a LabRAM HR800 with a 532 nm excitation laser (Horiba Jobin Yvon). The TRPL spectra were measured by a fluorescence spectrometer with a 478 nm excitation laser. (DeltaFlex, Horiba). The FT-IR spectra were measured by a Nicolet iS50R Fourier Transform Infrared Spectrometer (Thermo Scientific). The X-ray photoelectron spectroscopy (XPS)and ultraviolet photoelectron spectroscopy (UPS) spectra were measured by an Axis-Ultra DLD-600W X-ray photoelectron spectrometer (Shimadzu - Kratos), and the UPS spectra measurement used He I (hν = 21.22 eV) excitation. The *J-V* curves of space charge limited current (SCLC) was measured by a Keithley 2400 source/meter in the dark and recorded from 0 to 2 V with a scan rate of 100 mV/s. The electron-only devices with the structure FTO/SnO_2/perovskite/PCBM/Ag were fabricated for the measurement, where SnO_2 is tin oxide and PCBM is (6,6)-penyl-C61 butyric acid methyl ester. The defect density (N_t) of the perovskite films can be calculated by the following **Equation S1:**

$$N_t = \frac{2\varepsilon\varepsilon_0 V_{TFL}}{eL^2}$$

where ε is the relative dielectric constant of perovskites, ε_0 is the vacuum permittivity, V_{TFL} is the trap-filling limit voltage, e is the elementary charge, and L is the thickness of the perovskite film.

The *J-V* curves of devices were measured by a Keithley 2400 source/meter and a

Newport solar simulator (model 91192) that offered the simulated AM 1.5G illumination of 100 $mW \cdot cm^{-2}$, which was calibrated using NIST certified monocrystalline silicon solar cells (Newport 532 ISO1599). The active area of solar cells was defined by metal mask plate with an aperture of 10.1786 mm^2. The *J-V* curves were measured by a reverse scan (1.2 to −0.2 V) with a scan rate of 100 mV/s in the ambient condition. The light-dependent V_{OC} was obtained by testing the *J-V* curves of the devices at different light intensity. The test method and parameters are the same as above. Different light intensities were obtained by adjusting the grating of the Newport Solar Simulator (model 91192) and calibrated using a NIST-certified monocrystalline solar cell (Newport 532 ISO1599). The ideal factor can be obtained by fitting the following **Equation S2:**

$$V_{OC} = \frac{nkT_C}{q}\ln(I) + A$$

where k is Boltzmann' constant, T_C is the temperature of device, q is elementary charge, I is light intensity, A is a constant, n is the diode ideal factor. It is generally considered that n =1 represents a second-order (bimolecular) radiative recombination process and n =2 represents a first-order (unimolecular) nonradiative recombination process, such as defect-assisted non-radiative recombination[1].

The incident photo-to-current conversion efficiency was measured by a 150 W xenon lamp (Oriel) fitted with a monochromator (Cornerstone 74004) as a monochromatic light source. Capacitance–voltage (*C*–*V*) was performed with ZAHNER Zennium Electrochemical Workstation in dark with a voltage range from 0.1 to -1.2 V at a reserve scan direction with the AC perturbation of 10 mV and frequency of 20 kHz. The C-V data were analyzed by the following **Equation S3:**

$$C^{-2} = \frac{2(V_{bi} - V)}{A^2 q\varepsilon\varepsilon_0 N}$$

where C is the capacitance, V is the applied voltage, C is the built-in potential, A is the area of the perovskite, ε is the relative dielectric constant, ε_0 is the vacuum permittivity, N is the donor concentration of perovskite[2].

The Nyquist plots were measured with a ZAHNER Zennium Electrochemical Workstation in the frequency range of 100 mHz to 4 MHz without external bias under

dark condition.

Table S1 Detailed fitting parameters of TRPL of the perovskite films on m-ZrO_2.

Sample	τ_1 (ns)	τ_2 (ns)	A_1 (%)	A_2 (%)	T_{ave} (ns)
Control	6.72	209.99	0.77	0.23	52.73
3-CT	7.9	331.38	0.75	0.25	90.02
3-TEA	8.21	266.9	0.73	0.27	77.84
3-CT/3-TEA	8.66	418.46	0.69	0.31	136.25

Table S2 Detailed fitting parameters of TRPL of the perovskite films on m-ZrO_2/carbon electrode.

Sample	τ_1 (ns)	τ_2 (ns)	A_1 (%)	A_2 (%)	T_{ave} (ns)
Control	5.22	91.86	0.76	0.24	26.14
3-TEA	3.72	47.39	0.8	0.2	12.48

Table S3 Statistical performance parameters obtained from 60 devices.

Devices	V_{OC} (mV)	J_{SC} (mA·cm^{-2})	FF	*PCE* (%)
Control	950<br>(949.38 ± 12.83)	24.13<br>(22.3 ± 0.88)	0.712<br>(0.737 ± 0.016)	16.26<br>(15.60 ± 0.59)
3-CT	984<br>(984.94 ± 6.17)	24.11<br>(22.93 ± 0.89)	0.748<br>(0.759 ± 0.032)	17.76<br>(17.12 ± 0.22)
3-CT/3-TEA	1012<br>(994.67 ± 19.16)	24.21<br>(22.84 ± 0.85)	0.755<br>(0.778 ± 0.022)	18.49<br>(17.66 ± 0.34)

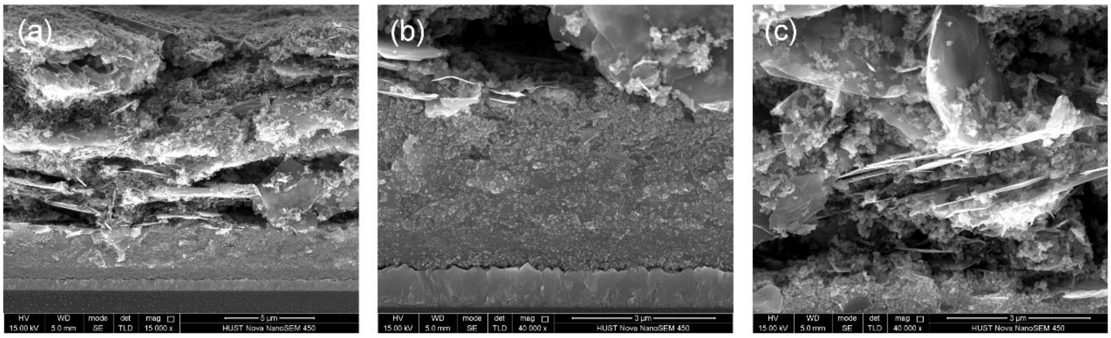

Figure S1 The cross-sectional SEM images of the printable mesoscopic PSCs (a) and local enlarged images of m-TiO_2/m-ZrO_2 (b) and carbon electrode (c).

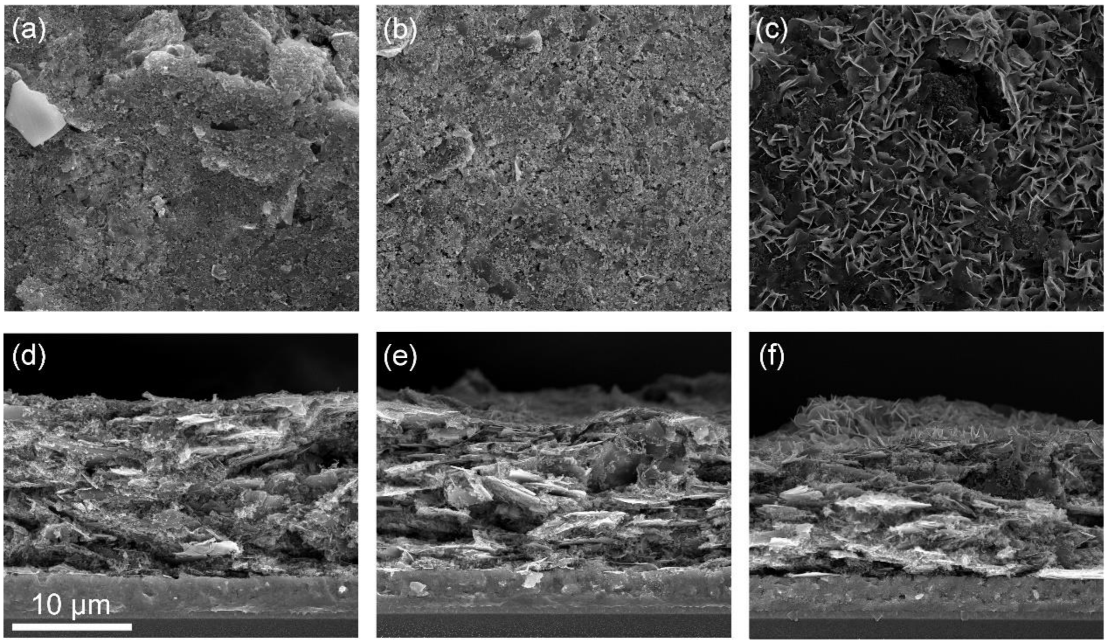

Figure S2 The Surface and cross-sectional SEM images of the control device (a, d), the 3-CT treated device (b, e), and the 3-TEA treated device (c, f).

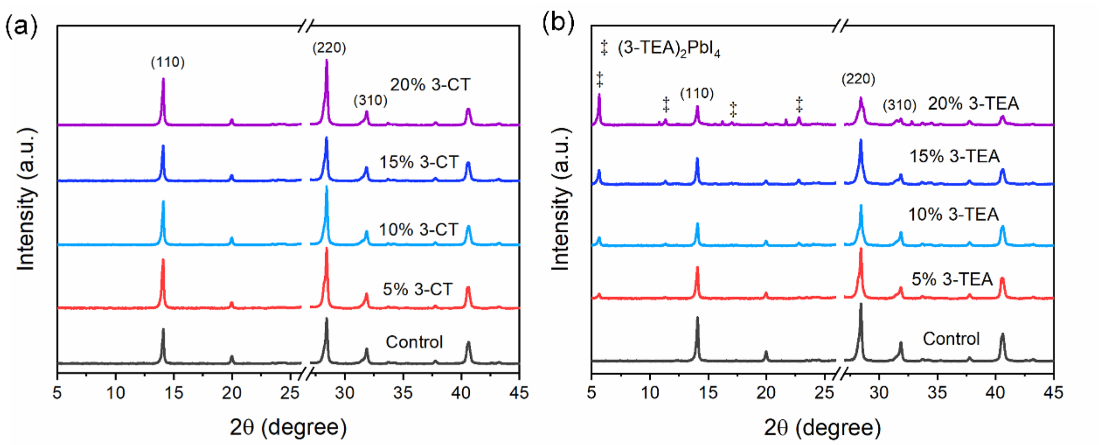

Figure S3 XRD patterns of the devices treated with different contents of 3-CT and 3-TEA.

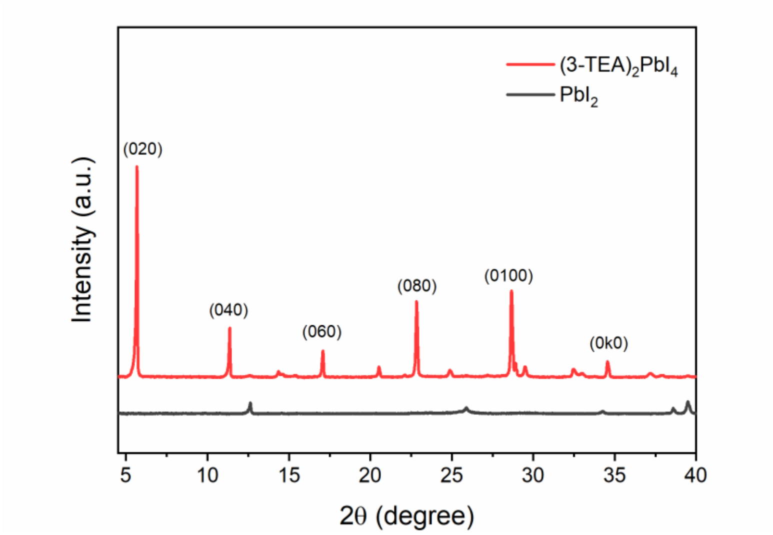

Figure S4 XRD patterns of the single crystal Ruddlesden-Popper $(3\text{-TEA})_2PbI_4$ perovskite.

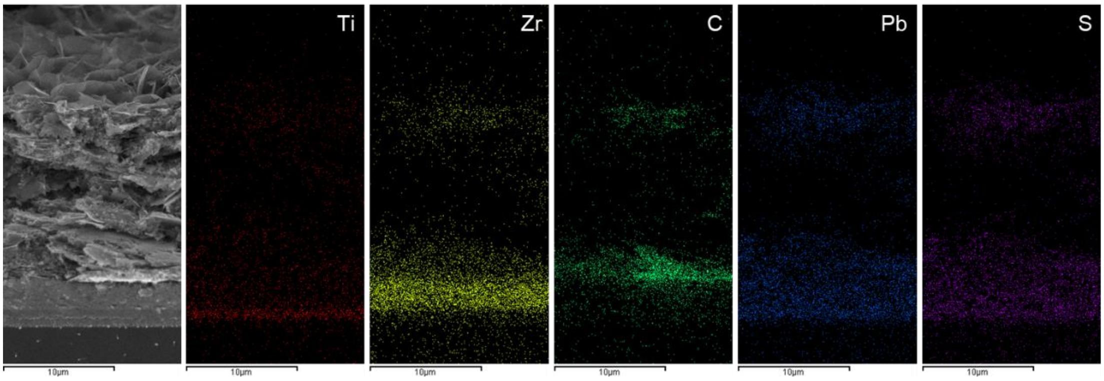

Figure S5 Cross-sectional scanning electron microscopy (SEM) of the 3-TEA treated device and energy-dispersive X-ray spectroscopy (EDX) analysis.

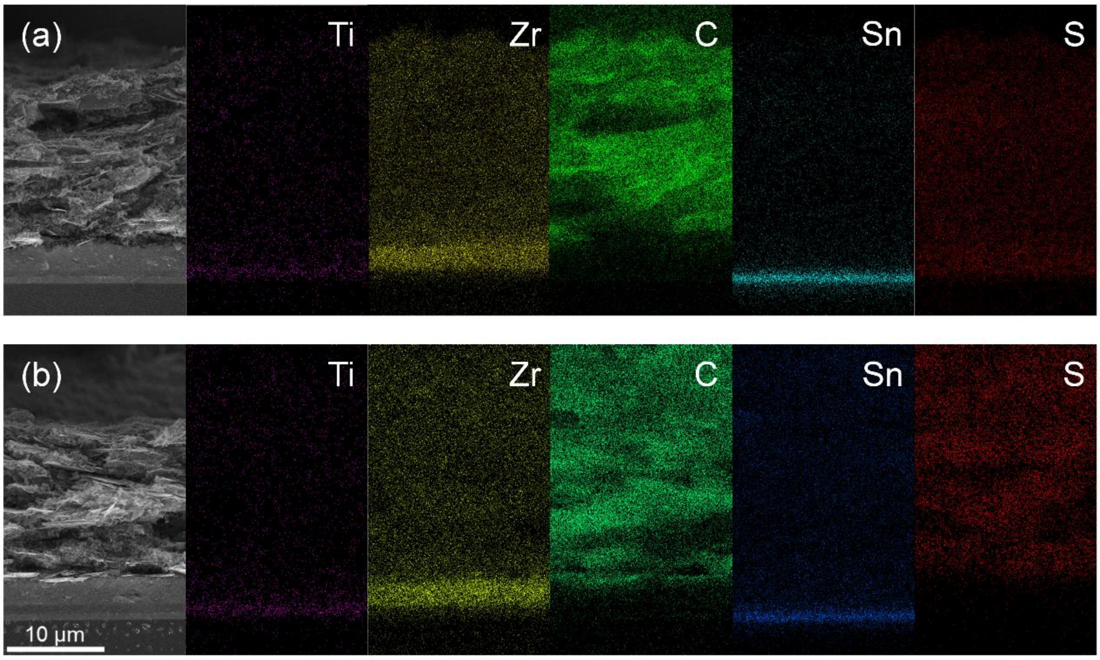

Figure S6 Cross-sectional SEM images of the 3-CT (a) and 3-TEA (b) treated devices based on $FASnI_3$ perovskite, and the corresponding EDX analysis. the Sn element represents the FTO and the $FASnI_3$ perovskite filled in the mesoscopic structure. The Ti, Zr and C element represents the m-TiO_2, m-TiO_2 and porous carbon electrode, respectively.

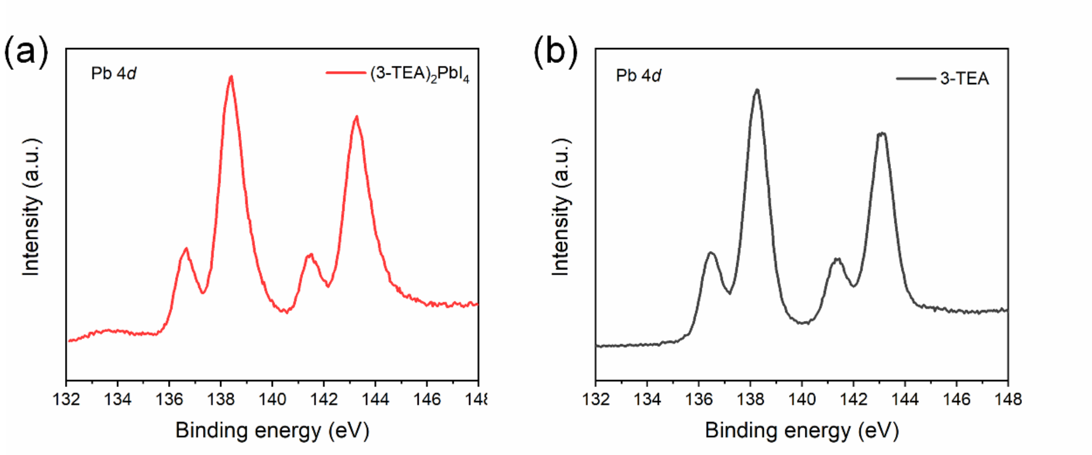

Figure S7 the X-ray photoelectron spectroscopy (XPS) of the $(3\text{-TEA})_2PbI_4$ perovskite (a), and 3-TEA treated $MAPbI_3$ perovskite (b).

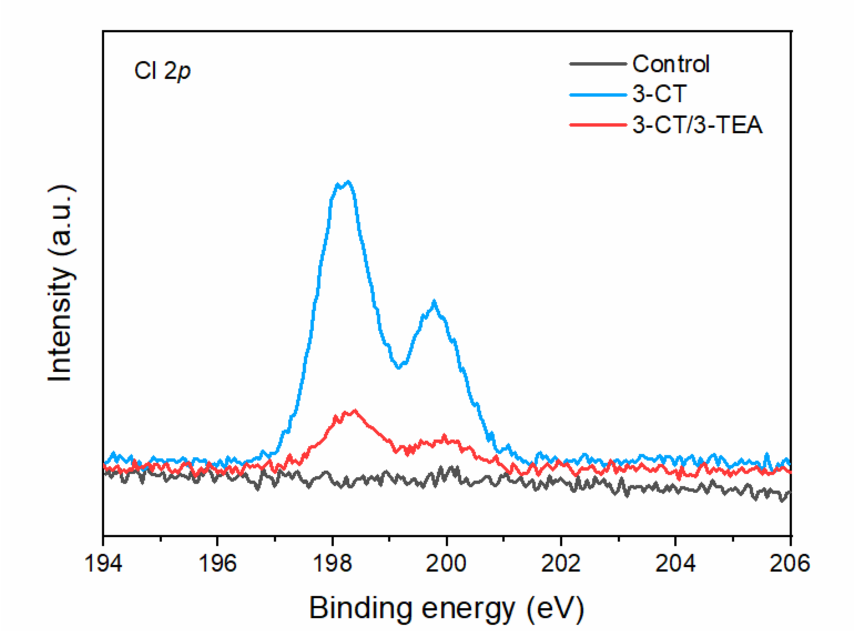

Figure S8 the high resolution XPS spectra of Cl 2*p*.

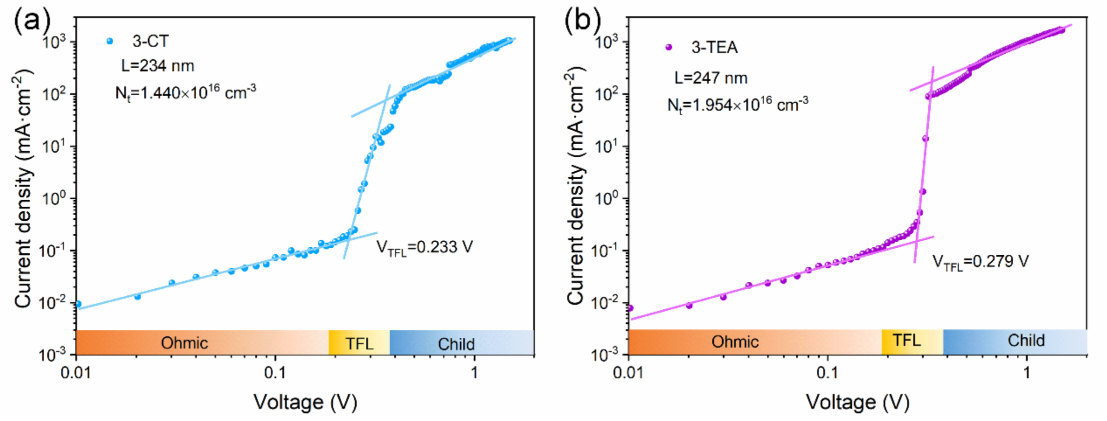

Figure S9 SCLC measurements of electron-only devices based on theMAPbI$_3$ treated with 3-CT (a) or 3-TEA (b).

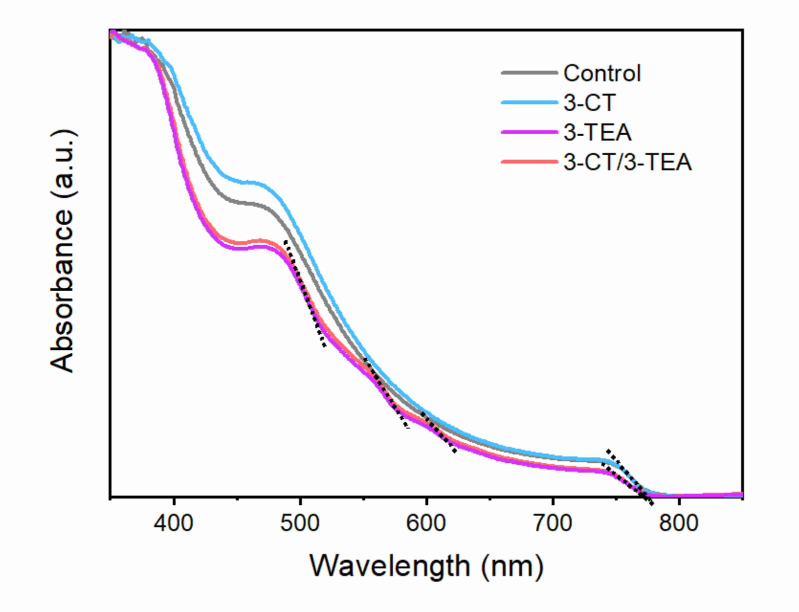

Figure S10 UV-vis absorption spectra of the perovskite films with the different molecular treatment.

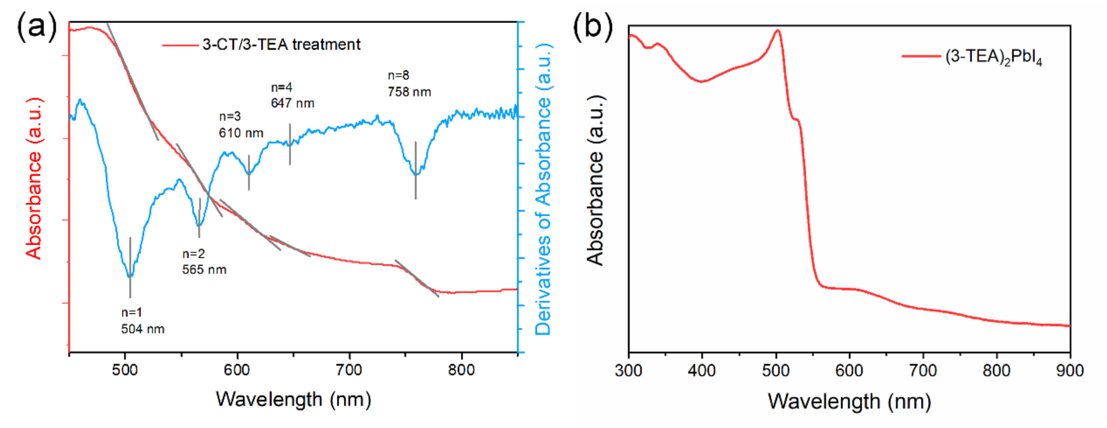

Figure S11 The UV-vis spectrum of 3-CT/3-TEA treated perovskite film (a) and 2D

perovskite $(3\text{-TEA})_2PbI_4$ (b).

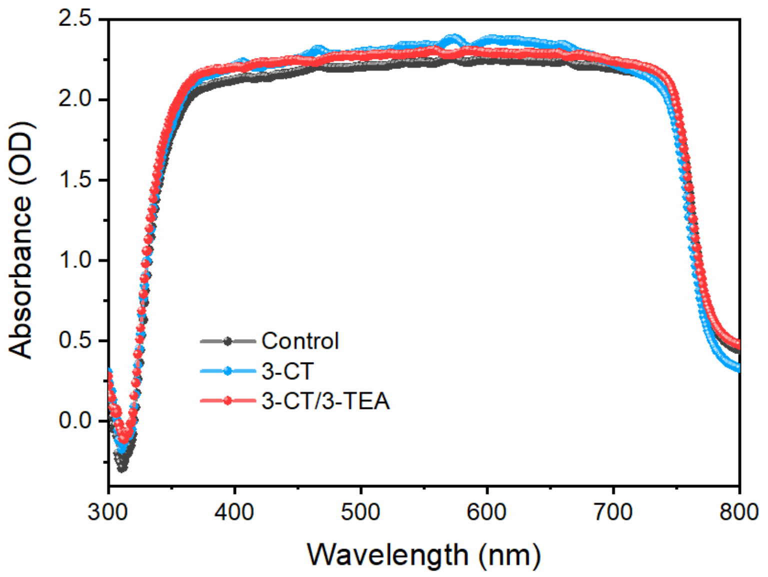

Figure S12 The UV-vis spectra (absorption pattern) of the different molecules treated devices stripped the carbon electrode. the absorbance of the 3-CT and 3-CT/3-TEA treated devices was not significantly different from that of the control, indicating that the 3-CT/3-TEA treatment did not impair the light absorption of the devices. In the printable mesoscopic devices, the light absorbing layer is the perovskite filled in m-TiO_2. Previously, we proved that the 2D perovskites in the device are mainly distributed in the C electrode. Therefore, the 3-CT/3-TEA treatment of the device does not affect the light absorption of the solar cell.

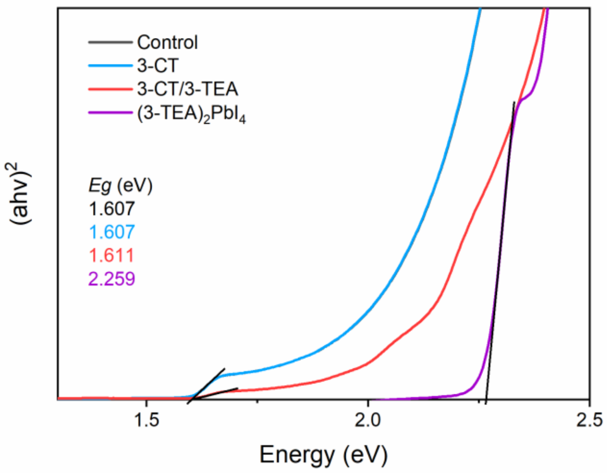

Figure S13 Tauc plots of control $MAPbI_3$, 3-CT and 3-CT/3-TEA treated $MAPbI_3$, and $(3\text{-TEA})_2PbI_4$ perovskites, and the corresponding fitted bandgaps are 1.607 eV, 1.607 eV, 1.611 eV, 2.259 eV, respectively.

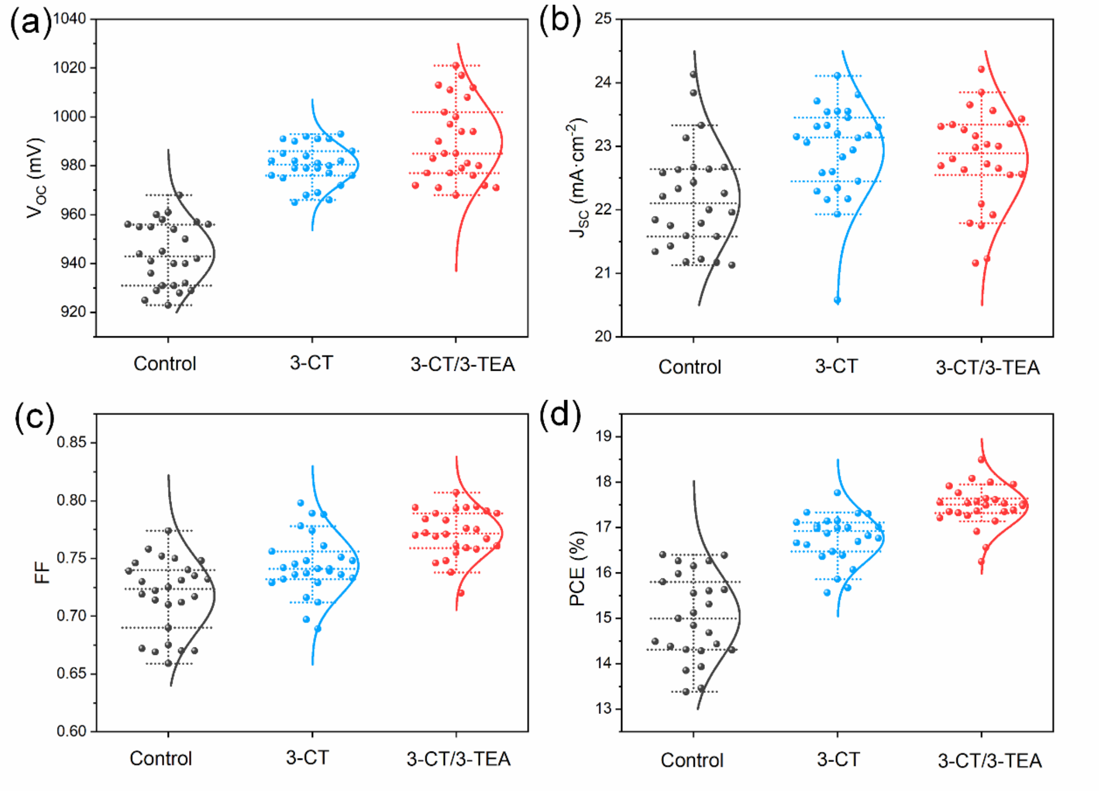

Figure S14 Statistical data for J_{SC}, V_{OC}, FF and *PCE* obtained from 75 devices treated with 3-CT and 3-CT/3-TEA.

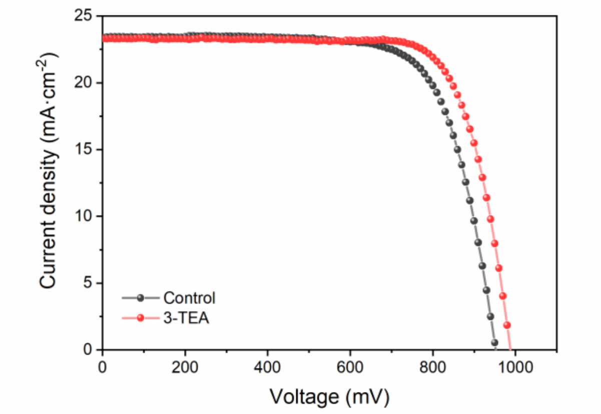

Figure S15 The *J-V* curves of the control and 3-TEA post-treated devices.

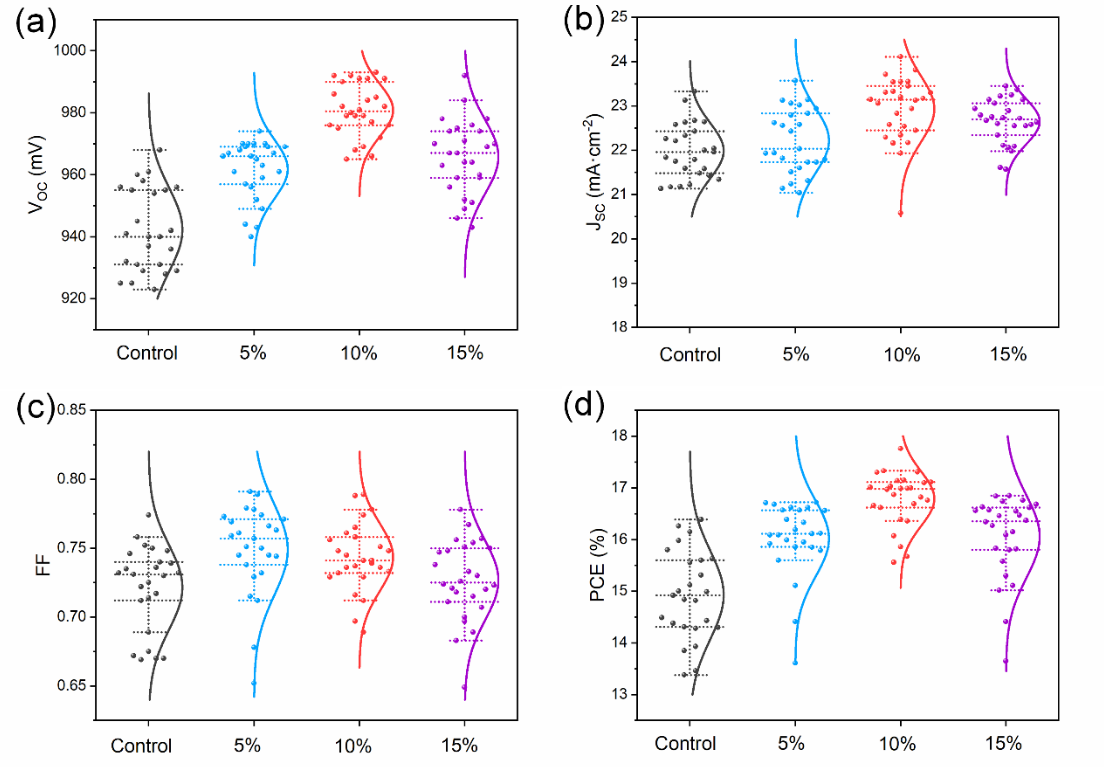

Figure S16 Statistical data for J_{SC}, V_{OC}, FF and PCE obtained from 100 devices treated with different percentages of 3-CT.

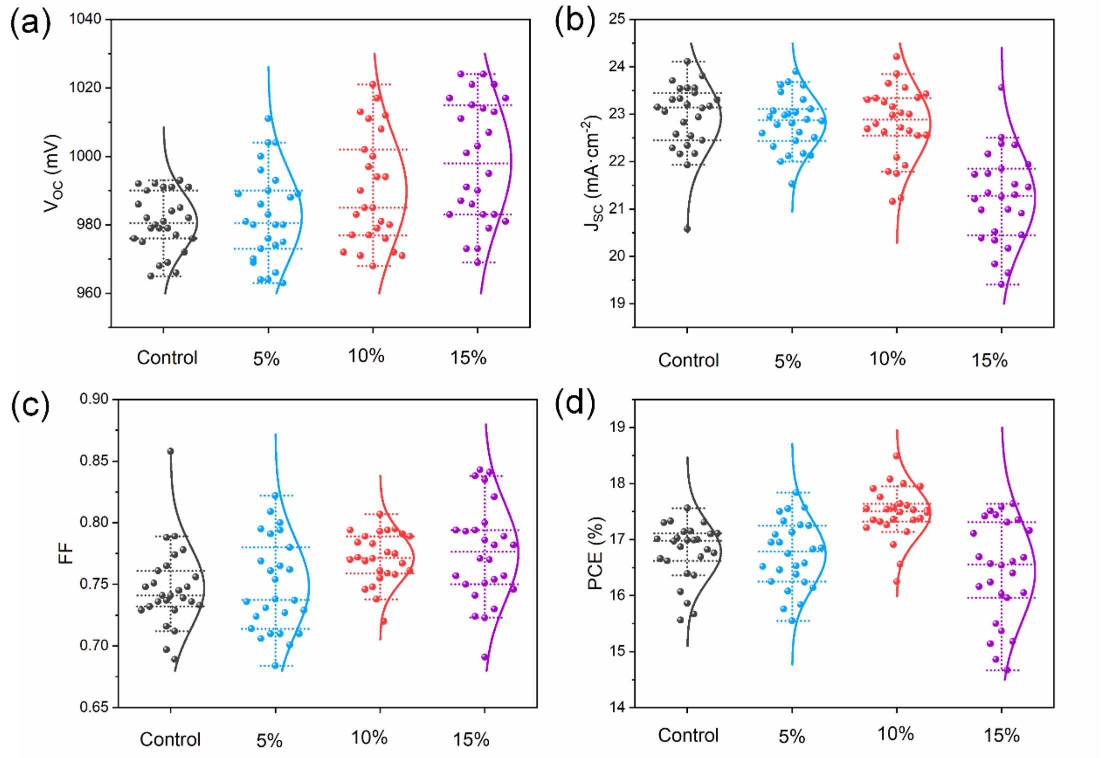

Figure S17 Statistical data for J_{SC}, V_{OC}, FF and PCE obtained from 100 devices treated with different percentages of 3-TEA on top of the 10% 3-CT treatment.

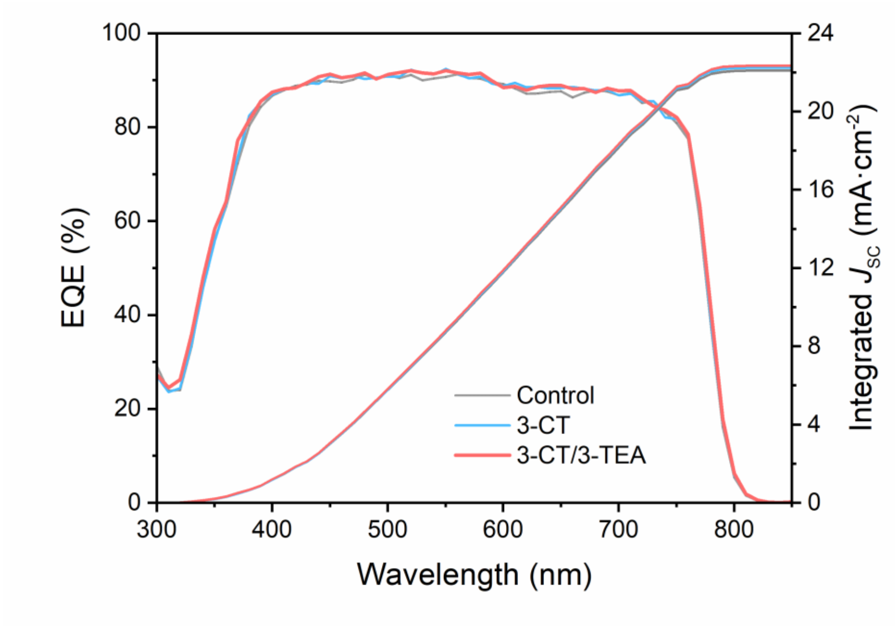

Figure S18 The incident photon-to-current efficiency (IPCE) spectrums of different molecules treated devices.

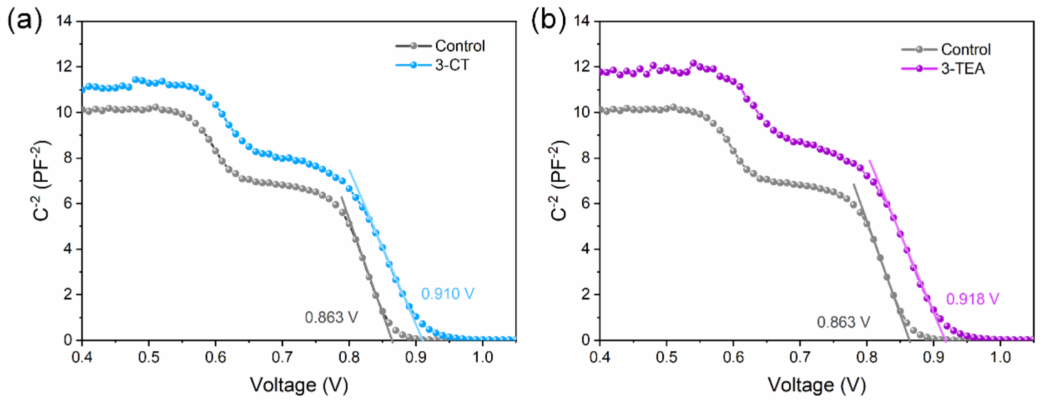

Figure S19 Mott−Schottky plots of 3-CT (a) and 3-TEA (b) treated devices.

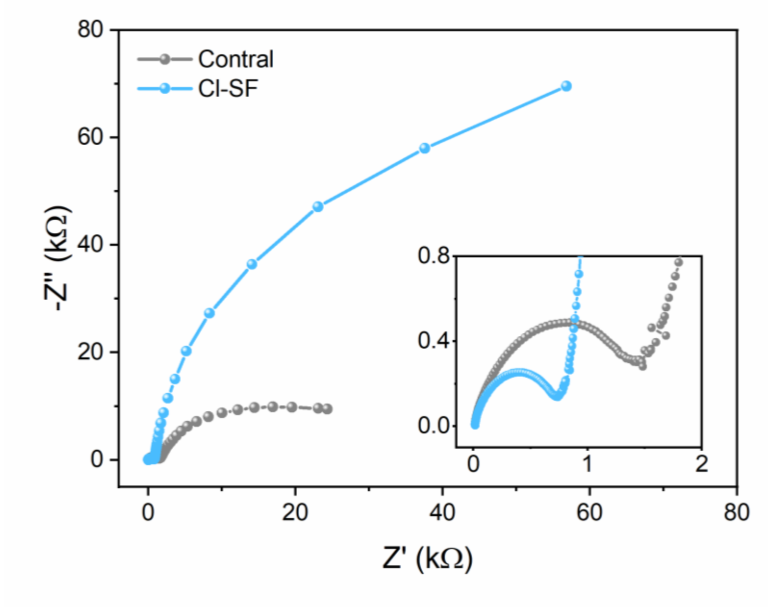

Figure S20 Nyquist plots at open circuit state of the 3-CT treated devices in the dark.

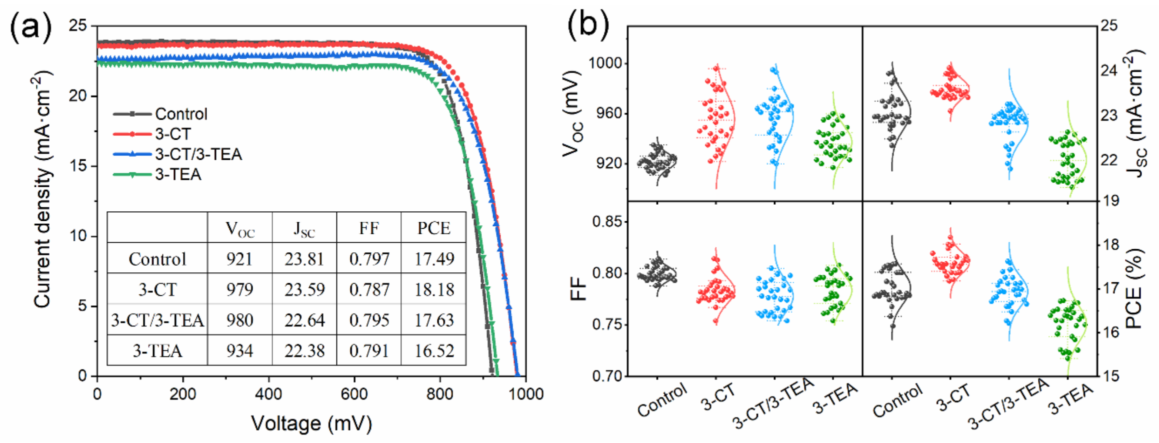

Figure 21 (a) The *J-V* curves of the champion devices based $Cs_{0.05}MA_{0.15}FA_{0.8}PbI_3$ perovskite. (b) Box plot of different devices performance parameters distribution and the statistical data were collected from 30 devices each.

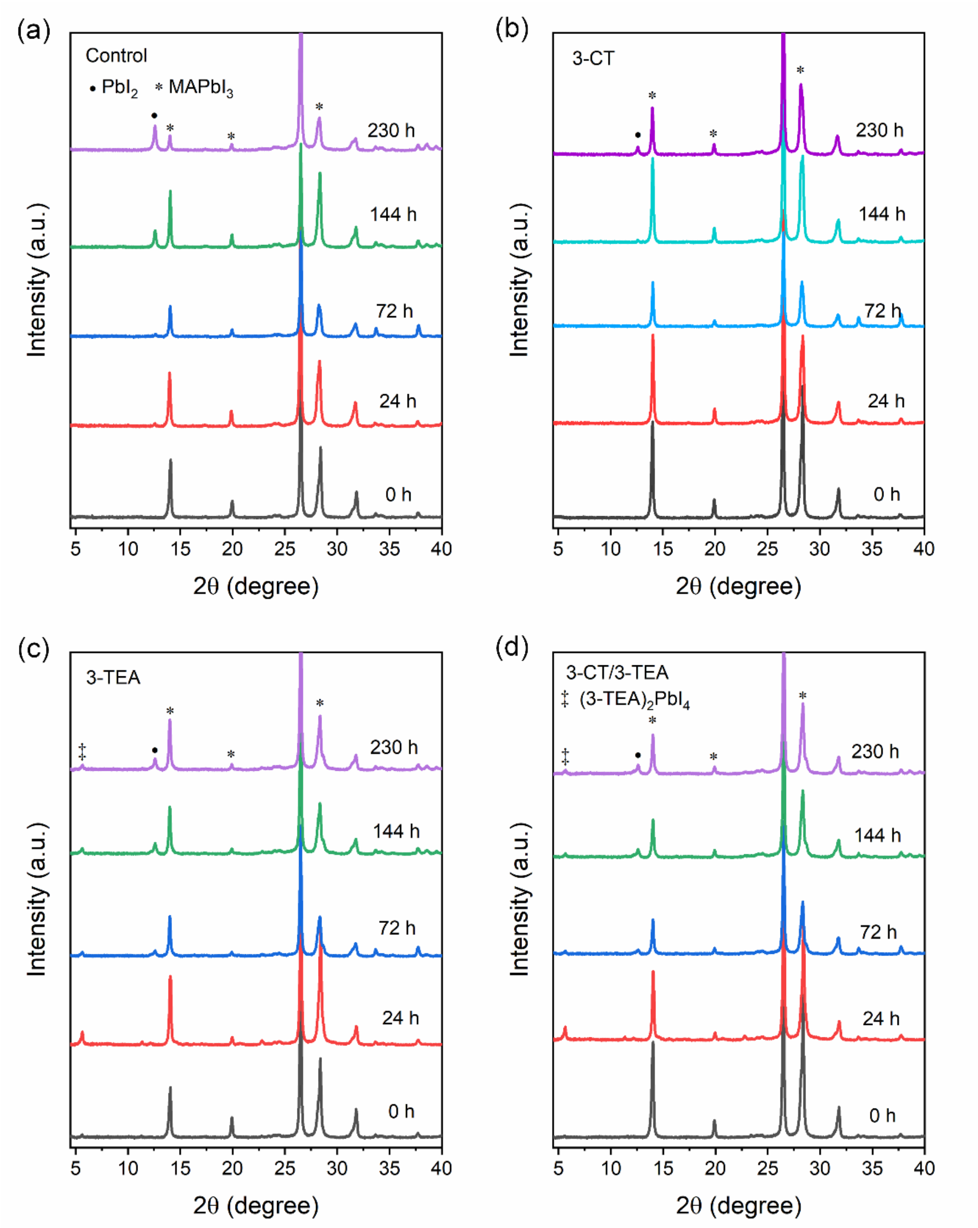

Figure S22 The XRD patterns of control, 3-CT, 3-TEA, and 3-CT/3-TEA treated devices with different storage durations at 85 °C in a N_2-filled glovebox.

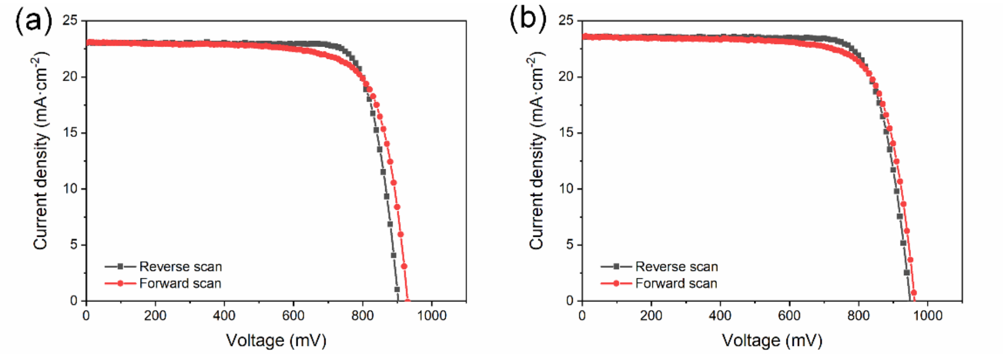

Figure S23. *J-V* curves of (a) the control device and (b) the device treated with 3-CT/3-TEA in reverse and forward scans.

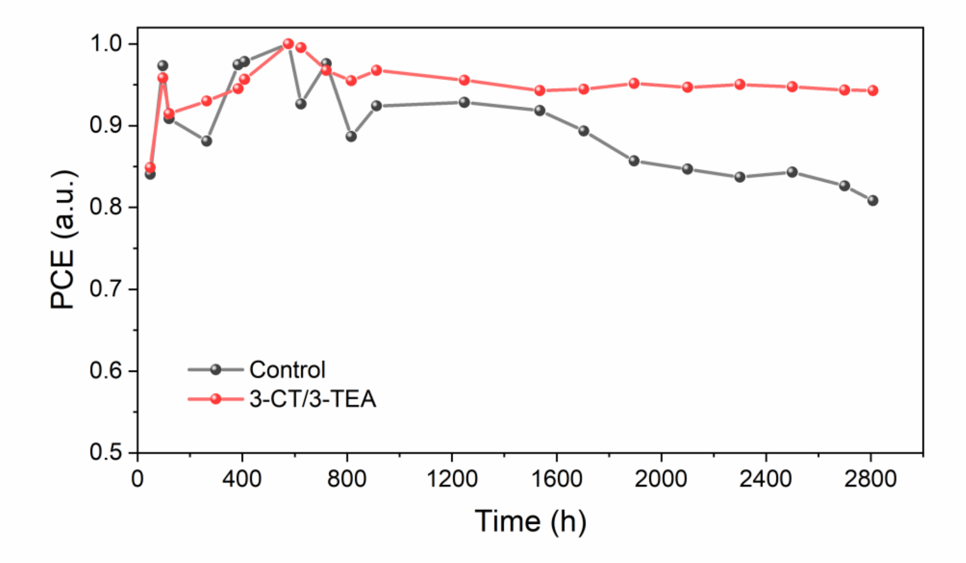

Figure S24 Storage stability of unpackaged devices. (Temperature: 25 ± 5°C, Humidity: 50 ± 20%RH).

References

[1] N. Shibayama, H. Kanda, T. W. Kim, H. Segawa, S. Ito, *APL Mater.* **2019**, 7, 031117.

[2] W. Wang, P. Chen, C. Chiang, T. Guo, C. Wu, S. Feng, *Adv. Funct. Mater.* **2020**, 30, 1909755.
